# Fellowships, Grants, & Awards

**Published:** 2007-07

## Behavioral and Social Science Research on Understanding and Reducing Health Disparities (R01)

The National Institutes of Health (NIH) issues this Funding Opportunity Announcement (FOA) to solicit research project grant applications (R01) employing behavioral and social science theories, concepts, and methods 1) to improve understanding of the causes of disparities in health and disability among the various populations of the United States and 2) to develop and test more effective interventions for reducing and eventually eliminating health disparities. The goal is to move beyond documenting the existence of health and disability disparities to addressing causes and solutions.

### Definition of health disparities

Different public and private agencies have various definitions of a health disparity for their own program-related purposes, but these definitions tend to have several things in common. In general, health disparities are defined as significant differences between one population and another. [See Office of Minority Health, U.S. Department of Health and Human Services (HHS), http://www.omhrc.gov/templates/content.aspx?ID=3559.] The Minority Health and Health Disparities Research and Education Act of 2000, which authorizes several HHS programs, describes these disparities as differences in “the overall rate of disease [or disability] incidence, prevalence, morbidity, mortality or survival rates as compared to the health status of the general population.” Although many different populations experience health disparities, this FOA is restricted to health disparities among populations defined by socioeconomic status, race/ethnicity, and/or rural–urban residence.

### Fuller understandings of causes and implications for solutions

Scientific research supported by NIH has been of great benefit to the health of the population in the United States. Research to improve diagnosis, treatment, and prevention has led to improvements in health care for most Americans, and significant declines in disability, morbidity, and mortality from numerous diseases and conditions. As a result, the population can expect not only to live longer, but to be more productive and to enjoy a higher quality of life. However, these gains have not affected all segments of the population equally. A few examples of persistent health disparities are as follows: 1) Over the last decade, the infant mortality rate remains more than twice as high among African Americans compared with European Americans, even when controlling for socioeconomic factors. American Indians and Alaskan Native infants also have a death rate almost double that of European Americans. 2) A disproportionate burden of death and disability from cardiovascular disease and stroke is found in low-income populations and among African Americans. 3) Incidence of reported lung, colon, rectal, and cervical cancer is substantially higher in the Appalachian region, where the incidence of lung and cervical cancer is one-third higher than the national average. African Americans have both a higher overall incidence and a higher death rate than any other racial or ethnic group. 4) The disease burden associated with mental disorders falls disproportionately on certain ethnic/racial populations. Native Americans and Alaska natives not only suffer disproportionately from depression, but also experience a higher rate of suicide. 5) Substantive differences exist in patterns of alcohol consumption and related consequences across a variety of racial and ethnic groups. For example, alcohol-related cirrhosis death rates are higher among white Hispanic males when compared with white non-Hispanic males, and the rate for black Hispanic males is lower than for either comparison group; and the frequency of alcohol-related traffic deaths is much higher among American Indians and Alaska natives compared with other ethnic/racial populations. 6) Epidemiologic data show little difference in overall drug use by race/ethnicity; yet there are great differences in consequences of drug use for racial/ethnic minorities, creating a great need to better understand the unique prevention, treatment, and health service needs of these communities. 7) The prevalence of osteoarthritis, hypertension, cervical cancer, and of all chronic diseases combined progressively decreases as socioeconomic status increases. 8) The prevalence of diabetes is greater in African Americans, Hispanic Americans, American Indians, certain Pacific Islanders, and Asian American populations, and in economically disadvantaged people than in the overall European American population. 9) Surveillance data indicate that 69% of new HIV infections are in individuals from racial and ethnic minority groups (i.e., African Americans, Hispanics, Native American/Alaska Natives, Asian/Pacific Islanders/Native Hawaiians) although these communities make up less than 25% of the U.S. population. 10) Disparities in the identification and treatment of communication disorders (i.e., disorders of hearing, balance/vestibular, smell, taste, voice, speech, and language) in diverse populations result in a disproportionate burden of these disorders among groups of lower socioeconomic status and selected racial/ethnic backgrounds. 11) Disparities exist in the prevalence of caries, oral and pharyngeal cancer, and periodontal disease for racial/ethnic minorities and the poor. Over one-half of Native American/Alaska Native and Hispanic children suffer from a devastating form of tooth decay—Early childhood caries. African-American males and subgroups of Hispanic male populations are at increased risk for late-stage malignancies. Edentulism is highest amongst Appalachians. Recent trend analyses indicate that disparities have increased for many disadvantaged populations.

In summary, U.S. populations defined by lower socioeconomic status, certain racial/ethnic backgrounds, and rural residence continue to experience substantial disparities in the burden of disease and death when compared with the U.S. population as a whole or to European Americans.

The research opportunities identified in this announcement are the result of discussions between the extramural research community and the NIH Behavioral and Social Sciences Research Coordinating Committee as well as at the NIH Conference on Understanding and Reducing Disparities in Health: Behavioral and Social Sciences Research Contributions (23–24 October 2006). This announcement highlights important areas for investigation that may not be included in the health-disparities strategic plans of individual NIH Institutes or Centers. (See Strategic Research Plan and Budget to Reduce and Ultimately Eliminate Health Disparities, FY 2002–2006, http://ncmhd.nih.gov/our_programs/strategic/volumes.asp.)

Given the extensive scientific literature documenting health disparities, this announcement calls for research to improve and elaborate explanations and understandings of the causes for health disparities. In so doing, the announcement stresses the explicit employment of concepts and models from the behavioral and social sciences to guide basic and applied research by focusing on three action areas: public policy, health care, and disease/disability prevention. (See below.) It emphasizes 1) basic research on the behavioral and social—acting with or through biological—pathways that give rise to disparities in health, and 2) applied or translational research on the development, testing, and delivery of interventions to reduce disparities. It encourages a multilevel analytic framework (i.e., ranging from individuals to societies) in investigating public health issues and their interactions (e.g., multiple morbidities rather than single illnesses) as well as attention to risk factors or causal processes common to various health conditions (e.g., smoking, diet, exercise, and access to health care).

Moreover, this announcement encourages research on the causes of and solutions to the “health differences” between a focus-population group and a reference-population group (e.g., African Americans vs. European Americans or the U.S. population as a whole). By definition, health disparities refer to the health of a group in comparison to the health of other groups. Although improving the absolute level of a population group’s health is a laudable goal, it may not result in changing the group’s relative level of health: The reference population’s health might also improve, thereby maintaining or even widening the gap. The study of a single population group (in order to elucidate the circumstances that may contribute to health disparities or to test an intervention targeting a particular group) may be included under this announcement. However, the relevance to disparities must be addressed explicitly. Also of interest is research on the causes of disparities within a single population group (e.g., among African Americans).

### Areas of action affecting health disparities

This FOA focuses on three broad areas of action influencing health disparities: public policy, health care, and disease/disability prevention. For the purposes of this FOA, these action areas are defined as:

#### Public policy

Public policy may be defined as the means employed by governments and other institutions to influence the function and well-being of individuals, groups, communities, and society as a whole. Some public policies at the national, state, and local levels are designed explicitly to affect health and may have direct impacts on health disparities. Examples include medical insurance programs for the elderly, disabled, and poor; alterations in health programs to contain costs; occupational safety regulations; and regulation of environmental hazards.

In addition, policies with no explicit health focus may also affect health and health disparities indirectly. For example, laws prohibiting discriminatory housing practices or housing subsidies may reduce health disparities by ameliorating exposures to environmental toxins among poor and ethnic/racial populations. The provision of child-care centers in workplaces may increase breast-feeding. Income maintenance programs may help to reduce stress and improve diets. Whereas such effects are plausible given known pathways linking socioeconomic disadvantage and racial/ethnic status to health, research evidence documenting their existence and strength is largely lacking.

Public and private sector policies are an important—and modifiable—element of the complex social environmental system that contributes to health disparities. Although scientific knowledge is rarely the only factor driving the formation of policies, rigorous and objective scientific studies can help to inform policy making by providing data on which to base assumptions about the costs and benefits—and likely effects—of various policy options.

Advancing knowledge about the potential for reducing health disparities through policy mechanisms requires a broad set of research studies, including both basic and intervention research. Further, interdisciplinary efforts are needed to bridge the many different kinds of economic, social, behavioral, and biological processes involved in translating public policy into public health. Examples include research on: 1) the interacting and cumulative effects on health disparities of policies formulated at a variety of levels—national, state, local, and nongovernmental; 2) innovative policy approaches to addressing pathways linking social and behavioral factors to health disparities, e.g., policies with the potential to build social capital in communities, alleviate stressors associated with disadvantage, or address targeted advertising of alcohol and tobacco in disadvantaged populations; 3) the behavioral and social mechanisms and processes linking policy to health disparities, including the role of social, cultural, and economic factors in mediating impacts and producing variations in policy implementation that affect outcomes; 4) knowledge transfer in the context of policy formation and implementation. (incorporating scientific knowledge with other kinds of information in the community, economic, bureaucratic, and legal processes that leads to the development of policies to affect health disparities); 5) cost-effectiveness of different policy strategies for addressing health disparities; 6) development of research designs and methodologies for studying policy effects on health disparities, including experimental, comparative, and other observational methods.

#### Health care

Health care is defined as the timely delivery of care and/or medical services by general or specialty providers to persons in need for the purpose of diagnosis, assessment, or treatment in order to improve or protect health status. Differences in the quantity and quality of health care targeted to and received by members of population groups are critical to understanding disparities in health.

For example, members of certain racial/ethnic groups are less likely than the general population to receive health care services. For example, blacks are less likely than whites to receive common diagnostic procedures and treatments or to receive intensive interventions such as bypass surgery. Furthermore, racial disparities exist in important qualitative aspects of medical care, such as receiving care from a private physician versus hospital outpatient or emergency departments.

Increased conceptual and empirical efforts are needed to identify and understand the processes leading to differentials in health care and to develop intervention strategies. Note that merely documenting or comparing utilization rates is not in the purview of this FOA. Explanatory analyses of the origins of differential rates or evidence-based interventions to improve rates are the focus of this FOA.

Disparities in the quantity and quality of health care may result from the interaction of several factors. Among these are: 1) Differential mix of health care services available to and accessible by various population groups. For example, physicians may tend to avoid areas with large racial/ethnic populations when establishing private practices; distances to health care services may be greater for those living in racial/ethnic communities; outreach and health promotion activities of agencies may be less effective. A related question is how the currently evolving health care system, such as HMOs, affects health disparities. 2) Inadequate economic resources or poverty-related factors (e.g., time constraints, lack of access to transportation, unsafe environments) may result in foregoing or postponing medical services. For example, Hispanic adults are substantially more likely to be uninsured than white or black adults. 3) Cultural, attitudinal, or communication-style differences between members of various populations and health-care providers may lead to mis-communication, misunderstanding, and deficiencies in health care. 4) Individuals from a racial/ethnic, rural, or low-SES population may express their disease symptoms in ways different from members of the general population, which may lead to errors in diagnoses and treatment. 5) Prejudice and discrimination may influence decisions about providing healthcare services. 6) Anticipated prejudice and discrimination may negatively affect clinical care relationships and result in delays in seeking care and/or poor adherence to provider recommendations.

#### Disease/disability prevention

Prevention research encompasses investigations designed to yield results directly applicable to identifying and assessing risk, and to developing interventions for preventing or ameliorating high-risk behaviors, the occurrence of disease/disorder/injury or progression of detectable but asymptomatic disease. Prevention research also includes research studies to develop and evaluate disease/disability prevention and health promotion recommendations and public health programs. Included is research on: 1) identification of modifiable risk and protective factors for diseases/disorders/injuries that may differ across populations; 2) development of population-appropriate methods, drawing upon behavioral and social science findings, for screening and identification of markers for those at risk for onset or progression of asymptomatic diseases/disorders, or at risk for adverse high-risk behaviors/injuries; 3) using behavioral and social science theory and findings to develop and evaluate individual- or group-level interventions to promote health of individuals or populations without recognized signs or symptoms of the target condition; 4) translation of proven effective prevention interventions in the general population into practice among populations that experience health disparities (also see http://grants.nih.gov/grants/guide/pa-files/PAR-07-086.html); 5) effectiveness studies that explicate behavioral and social factors related to the organization, management, financing, and adoption of prevention services and practices; and 6) methodological and statistical procedures for assessing risk and measuring the differential effects of preventive interventions among populations experiencing health disparities.

Although several interventions to improve health-enhancing behaviors in the areas of smoking, drinking, physical activity, and diet have been developed, most previous research has targeted easy-to-reach populations. The effectiveness of these interventions for vulnerable populations in diverse population groups is still undetermined. Although a variety of theoretical models (health belief model, theory of reasoned action, transtheoretical model and stages of change, etc.) have been developed to describe the process of health behavior change, still unknown is the relevance of different theories for changing particular behaviors in various populations. In addition to research on individual level behavior change, gaps remain in the development and testing of community level interventions for a diversity of communities experiencing health disparities. Also needed is research on personal, cultural, and institutional barriers to intervention availability, delivery, and effectiveness as a function of population-group membership, as well as the mechanisms of intervention that work best to prevent disease in population groups experiencing health disparities.

#### Crossing boundries

The boundaries between these three broad topics are arbitrary and permeable. For example, policy is often directed explicitly at health care or prevention. Similarly, how health care is provided influences prevention (and vice versa). These categories are provided as heuristics for organizing topics. Consequently, the NIH encourages research on topics falling within more than one of these categories or on the interplay among the categories.

### Research perspectives and themes

To achieve the twin goals of a more comprehensive understanding of the causes of health disparities and to design and implement effective interventions to reduce and ultimately eliminate health disparities, this FOA encourages the application of several research perspectives and themes. The NIH believes these approaches may move current research efforts to the next level of accomplishment. Applicants are not required to incorporate all of these themes into their research proposals.

### Interdisciplinary collaborations

Addressing health disparities requires a greater understanding of the full range of factors that determine health—biological, medical, behavioral, social, and environmental—and of their complex interrelationships. In many instances, a single research discipline is best suited to tackle specific health problems. However, it is increasingly recognized that particular problems cannot be adequately addressed within a single discipline, and instead require a more comprehensive approach. New discoveries and innovative solutions may become possible when researchers in different disciplines meet at the interfaces and frontiers of those disciplines to pool their diverse knowledge. Interdisciplinary collaborations refer to scientific endeavors in which a variety of disciplines work together closely from the outset to form a shared conceptual framework to address a problem. Interdisciplinary research is distinct from multidisciplinary research in that the latter refers to a process in which researchers in different disciplines work relatively independently, each from his or her own disciplinary perspective with limited direct interaction and little cross-fertilization among disciplines. The NIH encourages interdisciplinary studies that cross the traditional boundaries within and between biological, behavioral, and social sciences.

#### Levels of analyses

A variety of scientists have offered the concept of levels of analysis to capture the distinct but interdependent levels at which health, and the determinants of health, can be understood. (See http://grants.nih.gov/grants/guide/pa-files/PA-05-029.html.) One schema identifies five major levels of analysis in health research: social/environmental, behavioral/psychological, organ systems, cellular, and molecular. Most research focuses on a specific level, which roughly corresponds to the domain of specific scientific disciplines. However, although the disciplines concerned with health research may be separated conceptually, methodologically, and administratively, the processes about which they are concerned are inextricably linked. A levels-of-analysis approach offers a framework for understanding the interdependence among levels. A variety of conceptual models exist to address the linkages among levels of analysis, from the macrosocietal levels to the biology of a disease, but they have not been uniformly accepted or systematically applied in empirical studies of health. One framework links social structure or social position (e.g., class, age, gender, race, ethnicity), environmental context or place (e.g., geographic location, housing conditions, access to services), lifestyles (e.g., smoking, physical activity), and physiology (e.g., blood pressure, cholesterol, obesity). Others suggest a metaphor of “Chinese boxes” to guide a new eco-epidemiology “which treats relationships within and between localized structures that are bounded socially, biologically, or topographically.” Such frameworks help to guide the development of multi-level research. They also illustrate how such research can inform public knowledge about health policy, organizational- and community-level interventions, and primary and secondary intervention. Thus, models that integrate, for example, factors operating at the social and cultural levels with those operating at the psychological and biological levels are especially encouraged. Many multilevel studies are also multimethod studies that integrate quantitative and qualitative data and thus strengthen measurement validity while retaining the capacity for statistical inference.

An accumulated body of empirical findings has clearly demonstrated that social and cultural factors create conditions of life that can protect or damage health. These conditions influence health by affecting such things as exposure and vulnerability to disease, risk-taking behaviors, the effectiveness of health promotion efforts, and access to, availability of, and quality of health care. They play a critical role in shaping individuals’ responses to health problems and influence how poor health affects individuals’ lives and well-being. The social sciences contribute to the nation’s health research agenda by addressing the dynamics of these social and cultural processes and the mechanisms through which they affect health.

A concern for health at the population rather than the individual level underscores the need to take social and cultural processes into account. An understanding of current and changing population rates of morbidity, survival, mortality, and use of health services requires that we consider the demographic, social, economic, and cultural features of the population. Needed is the investigation of the social, economic, and cultural systems as well as the individuals who participate in them.

#### Systems science methodologies

“Systems thinking” refers to bringing a perspective to problem solving in which the problem space is conceptualized as a system of interrelated component parts. The system is viewed as a coherent whole. The relationships among the components are also recognized and seen as critical to the system, for they give rise to the “emergent” properties of the system. Emergent properties are those properties that can only be seen at the system level and are not attributes of the individual components themselves (e.g., a flock emerges when a group of birds flies together; it is a property of the system, not of any individual bird). Systems approaches offer insights into the nature of the whole system that often cannot be gained by studying the component parts in isolation. Moreover, a systems approach recognizes that embedded in the system are feedback loops, stocks and flows, that change over time (i.e., dynamic complexity of the system). Advantages of using systems approaches as a complementary method for addressing complex problems include the fact that nonlinear relationships, unintended effects of intervening in the system, and time-delayed effects are often missed with traditional reductionistic approaches, whereas systems approaches excel at detecting these.

Systems approaches are able to address a broad range of factors within a single framework—from genetic to environmental, cellular to behavioral, and biological to social levels of analysis. Systems thinking is also logically related to knowledge and computing infrastructures necessary to link networks of researchers in their collaborative work. Successful application of these approaches in defense, business, and cellular biology have resulted in a growing interest in the use of systems approaches to population health research. The belief is that a systems approach shows promise for understanding and intervening on the complex, multidimensional relationships underlying health disparities.

A wide variety of methodologies are encompassed under systems science. Any variety is acceptable under this PAR. Here are some examples of the methodologies being sought under this PAR; note that this list is meant to be illustrative and not exhaustive: 1) agent-based modeling; 2) system dynamics simulation; 3) network analysis, including social network analytic methods; 4) discrete event simulation; 5) Markov modeling; 6) stochastic modeling; 7) differential equation/compartmental modeling.

Applicants are encouraged to learn more about systems methodologies and their role in behavioral and social science research at NIH by visiting the OBSSR Systems Science and Health webpage at http://obssr.od.nih.gov/Content/About_OBSSR/Activities/Systems_Science/.

#### Life-course perspective

Cumulative processes over the life course across multiple life domains at the individual and community levels are of central importance for understanding the associations between membership in socially defined population groups and health. For example, racial/ethnic group status influences early life conditions, including the fetal environment, that may be linked with later life expectancy and disease risks. Consequently, integrated investigation of psychosocial and physiological interrelationships over the life course and at critical developmental transitions are required in order to more fully understand the contemporaneous and cumulative impact of differential life experiences that underlie health disparities. Specifically, normative transitions (e.g., birth of a child, beginning school, emerging adulthood, retirement) often represent periods of increased vulnerability to both mental and physical health problems, and as such offer unique opportunities for intervention. For example, the early adolescent period has been identified as one which involves a combination of biological (e.g., puberty), social (e.g., increased role of the peer group), ecological (e.g., middle school), and cognitive (e.g., increased capacity for abstract thinking) changes as well as increased risk for certain disorders such as depression. Yet our understanding of the role of such developmental processes in the emergence, maintenance and potential alleviation of health disparities is limited. Thus, focusing attention on a wide variety of transitions across the lifespan along with the risk and protective factors related to them is needed for the ultimate development and testing of innovative interventions that target high risk periods across the life course. Such an approach emphasizes the fact that early life disadvantage and adversity need not lead to later negative outcomes, provided there are compensating positive experiences in the intervening years. Similarly, attention should be given to the positive aspects of people’s lives (e.g., positive social relationships and social support, education) that may buffer or compensate for the effects of adversity.

#### Community-based participatory research (CBPR)

CBPR is defined as scientific inquiry conducted in communities and in partnership with researchers. The process of scientific inquiry is such that community members, persons affected by the health condition, disability or issue under study, or other key stakeholders in the community's health have the opportunity to be full participants in each phase of the work (e.g., from conception, design, conduct, analysis, interpretation, conclusions to communication of results). CBPR is characterized by substantial community input in the development of the grant application (http://www.niehs.nih.gov/translat/cbpr/cbpr.htm).

Community-partnered approaches to research promise to deepen our scientific base of knowledge in the areas of health promotion, disease/disability prevention, and health disparities. (See http://grants.nih.gov/grants/guide/pa-files/PAR-07-283.html.) Community-partnered research processes offer the potential to generate better-informed hypotheses, develop more effective interventions, and enhance the translation of the research results into practice.

#### Prejudice and discrimination

Disparities in health exist for many reasons, but prejudice and discrimination—intentional and conscious as well as unintentional and unconscious—on the basis of race, ethnicity, sex, social class, sexual orientation, etc., appear to contribute significantly to differences in health care. (Also see http://grants.nih.gov/grants/guide/pa-files/PA-07-206.html.) For example, a recent study of racial factors that contribute to differentials in diagnosis and treatment demonstrated that racial bias is a significant influence on the likelihood that cardiac catheterization will be recommended for patients with chest pain.

Bias, discrimination, and prejudice are hypothesized to contribute to disparities in health through increased exposure and susceptibility to: 1) economic and social deprivation; 2) toxic substances and hazardous conditions; 3) socially inflicted mental and physical trauma, either directly experienced or witnessed; 4) targeted marketing of potentially harmful commodities such as tobacco, alcohol, illicit drugs; and 5) inadequate or degrading medical care.

The influence of actual as well as perceived (e.g., “stereotype threat”) prejudice and discrimination is not limited to access to health care. They can be sources of acute and chronic stress, which have been linked to conditions such as cardiovascular disease and alcohol abuse. Discrimination can restrict the educational, employment, economic, residential, and partner choices of individuals, affecting health through pathways linked with what psychosocial scientists refer to as “human” or “social” capital. Environmental influences from industry, toxic waste disposal sites, and other geographic aspects linked with poverty and racial/ethnic status can result in serious disadvantages to a population groups' health.

Evidence is insufficient to evaluate the magnitude of the relationship among prejudice, discrimination, and health. In addition, much of the empirical work investigating the effects of prejudice and discrimination and health has focused on African Americans. Few studies have addressed systematically how prejudice and discrimination affect other racial/ethnic groups such as Native Americans, Asian Americans, and Latinos or other socially defined populations. Prejudice and discrimination have helped shape the social position of each racial and ethnic group in the United States and, consequently, they may have unique associations with health for each group. Finally, an insufficient focus on the impact of societal forces has hindered our ability to understand and effectively address the influence of prejudice and discrimination on health disparities. The growing evidence that health, socioeconomic status, and macroeconomics are inextricably linked emphasizes the importance of undertaking a program of research to examine the relative magnitude of the influence of bias in the context of the other factors thought to affect racial/ethnic health.

#### Social context

The social environments in which processes affecting health and health disparities play out are often referred to as social context. These include familial, demographic, economic, political, legal, organizational, physical environmental, and cultural factors that affect the resources available to individuals throughout their life course. Applicants are encouraged to conceptualize and measure social contexts in order to specify which particular aspects of social context are factors in the production or maintenance of the health disparity under examination. They are also encouraged to conceptualize and measure the social processes that operate within and across social contexts and between social contexts and individuals.

Social context can be roughly divided into interrelated domains: families and households; social networks; neighborhoods; formal institutions; and public policy. Economic, social, and cultural processes interweave all of these domains. 1) Family structure, family resources, and family processes influence health across the life course. Families are centrally important for child health and development, influencing outcomes through parenting, adequate nutrition, obtaining health care, instilling healthy behaviors, and providing education and financial resources. Throughout life, families tend to operate as economic units and provide social, emotional, and instrumental supports (or create interpersonal stresses) that influence health and health-related behaviors. Socioeconomic status includes income, education, employment opportunities, and job characteristics. Family financial status affects the ability to live in a safe and healthy environment, and to provide members with a variety of goods and services—including medical care and nutrition—that affect their health. The educational levels of adults in a family are related to health knowledge (e.g., the ability to follow medical protocols) and behaviors (e.g., smoking and drug use), that subsequently affect other family members such as children. Family processes reflect cultural understandings and also imbue or reinforce them in family members. For example, family racial or ethnic identity is played out in family interactions and rituals, and may provide a source of resilience in the face of discrimination and stress. Family values regarding appropriate responses to disease symptoms are reinforced in parenting practices and affect the timing of health care seeking. 2) Social networks are defined as a web of social ties that connect people to others. Social networks provide individuals and their families with social support that may come in the form of emotional support that buffers individuals from poor physical or mental health, or in the form of information or instrumental help that can be used to maintain or improve health. Persons living in large, urban high-rise housing developments with little social organization and community support or in sparsely populated rural areas may be at a disadvantage for developing supportive social networks. Social networks also channel the diffusion of ideas and practices; they are the nexus for the creation of cultural norms and beliefs. They therefore may play a vital role in community-based interventions that depend on the spread of new ideas for their success. 3) Neighborhoods and communities provide resources that are important for the health of its members. These resources include the level of income in the community, the quality of community organizations and formal institutions, and employment opportunities. Social processes that determine the degree of social interaction, crime levels, and political activity characterize communities. Structural characteristics of neighborhoods, such as age, racial and ethnic composition, population density, and housing stocks, have an impact on social processes and the resources available to neighborhood residents. Racial/ethnic and poor communities are disproportionately exposed to health-damaging physical environments characterized by overcrowding, noise, substandard housing, insufficient public services, and toxic chemicals (including air pollution). A close connection exists among the physical, built, and social environments within neighborhoods and communities. For example, communities that have higher incomes and more effective community and political organizations may be better positioned to create and maintain physically healthy environments. 4) Finally, formal institutions (e.g., institutions, including schools and child care facilities, recreational facilities, law enforcement and justice programs, social services, religious institutions, and the media) affect the health of individuals and populations within a community. For example, poorly functioning institutions provide inadequate services and diminish the social capital of communities. Formal institutions are central to this announcement: they create and implement policies, design and operate prevention programs, and provide health services.

### Examples of research topics

Several NIH components and the Centers for Disease Control and Prevention (CDC) have joined together to support this FOA. Applications should be relevant to both the objectives of the FOA and to at least one of the participating organization's research interests. Researchers are strongly encouraged to review the general research interests of the participating organizations and the examples of topics of interest specific to health/disabilities disparities, which are posted at http://obssr.od.nih.gov/Content/Health_DisparitiesPAR_R01.htm.

This FOA will use the NIH Research Project Grant (R01) award mechanism.

The applicant will be solely responsible for planning, directing, and executing the proposed project.

This FOA uses “Just-in-Time” information concepts. It also uses the modular as well as the nonmodular budget formats (see http://grants.nih.gov/grants/funding/modular/modular.htm). Specifically, if you are a U.S. organization and are submitting an application with direct costs in each year of $250,000 or less (excluding consortium Facilities and Administrative [F&A] costs), use the PHS398 Modular Budget component provided in the SF424 (R&R) Application Package and SF424 (R&R) Application Guide (see specifically Section 5.4, “Modular Budget Component,” of the Application Guide).

U.S. applicants requesting more than $250,000 in annual direct costs and all foreign applicants must complete and submit budget requests using the Research & Related Budget component found in the application package for this FOA. See NOT-OD-06-096, 23 August 2006.

At this time, it is not known if competing renewal (formerly “competing continuation”) applications will be accepted and/or if this FOA will be reissued.

Applicants must download the SF424 (R&R) application forms and the SF424 (R&R) Application Guide for this FOA through Grants.gov/Apply.

Note: Only the forms package directly attached to a specific FOA can be used. You will not be able to use any other SF424 (R&R) forms (e.g., sample forms, forms from another FOA), although some of the “Attachment” files may be useable for more than one FOA.

For further assistance, contact GrantsInfo, 301-435-0714, (telecommunications for the hearing impaired: TTY 301-451-0088) or by e-mail: GrantsInfo@nih.gov.

The letter of intent receipt dates for this PAR are 20 August 2007, 2008, and 2009, with the application receipt dates 19 September 2007, 19 September 2008, and 18 September 2009. The complete version of this PAR is available at http://grants.nih.gov/grants/guide/pa-files/PAR-07-379.html.

Contacts: The complete list of agency contats is available at http://grants.nih.gov/grants/guide/pa-files/PAR-07-379.html.

Reference: PAR-07-379.

